# Cervical Cancer Screening Among Women Who Gave Birth in the US-Mexico Border Region, 2005: The Brownsville-Matamoros Sister City Project for Women’s Health

**Published:** 2008-09-15

**Authors:** Brian C Castrucci, Alonso Echegollen Guzmán, Mona Saraiya, Steven S Coughlin, Kayan L Lewis, Ginger L Gossman, Hillary Foulkes, Gita Mirchandani, Brian R Smith, Jill A. McDonald, Juan Acuña, Luz Correa-Nieto Canedo, Imelda M Garcia

**Affiliations:** Texas Department of State Health Services; Mexican Institute of Social Security, Tamaulipas, Mexico; Division of Cancer Prevention and Control, National Center for Chronic Disease Prevention and Health Promotion, Centers for Disease Control and Prevention (CDC), Atlanta, Georgia; Division of Cancer Prevention and Control, National Center for Chronic Disease Prevention and Health Promotion, Centers for Disease Control and Prevention (CDC), Atlanta, Georgia; Office of Title V, Division of Family and Community Health Services, Texas Department of State Health Services, Austin, Texas; Office of Title V, Division of Family and Community Health Services, Texas Department of State Health Services, Austin, Texas; Office of Title V, Division of Family and Community Health Services, Texas Department of State Health Services, Austin, Texas; Office of Title V, Division of Family and Community Health Services, Texas Department of State Health Services, Austin, Texas; Health Service Region 11, Division of Regional and Local Health Services, Texas Department of State Health Services, Harlingen, Texas; Division of Reproductive Health, National Center for Chronic Disease Prevention and Health Promotion, CDC, Atlanta, Georgia; Division of Reproductive Health, National Center for Chronic Disease Prevention and Health Promotion, CDC, Atlanta, Georgia; Cytology Department, State Reference Laboratory, Health Ministry, Tamaulipas, Mexico; Community Health Services Section, Division of Family and Community Health Services, Texas Department of State Health Services, Austin, Texas

## Abstract

**Introduction:**

The objective of this study was to examine correlates of ever having had a Papanicolaou (Pap) test among women who recently delivered a live infant and who resided near the US-Mexico border.

**Methods:**

This cross-sectional study included women who delivered a live infant in Matamoros, Mexico (n = 488) and Cameron County, Texas (n = 453). Women were interviewed in the hospital before discharge between August 21 and November 9, 2005. Multivariable logistic regression was used to estimate the odds of ever having had a Pap test.

**Results:**

Significantly fewer Matamoros women (62.1%) than Cameron County women (95.7%) reported ever having had a Pap test. Only 12% of Matamoros women said they received their most recent Pap test during prenatal care, compared with nearly 75% of Cameron County women. After adjusting for potential confounders, the odds of ever having had a Pap test were 7.41 times greater in Cameron County than in Matamoros (95% confidence interval, 4.07-13.48).

**Conclusion:**

The *Healthy Border 2010* goals are to cut cervical cancer mortality by 20% to 30% in the border region. The significant difference in Pap test prevalence among our survey respondents may reflect that routine prenatal Pap testing is more common in the United States than in Mexico. Because women who are receiving prenatal care have increased interaction with health care providers, Matamoros providers may need to be educated about the need to screen for cervical cancer during this time.

## Introduction

Cervical cancer incidence and mortality rates are higher among women in Mexico (29.5 per 100,000 and 14.1 per 100,000, respectively) than among Hispanic women in the United States (12.2 per 100,000 and 3.1 per 100,000) (1-3). Cervical cancer remains the leading cause of cancer deaths among women in Mexico, accounting for 16.5% of all cancer deaths among women, compared with 2.4% in the United States (4).

In the United States, the successful implementation of the Papanicolaou (Pap) test to screen for precursor lesions has reduced both the incidence of and mortality from cervical cancer in the last 50 years (5,6). Despite the initiation of a national cervical cancer screening program in Mexico in 1974, screening rates vary from 15% in very rural areas to 64% in urban areas (7-9). Two previous studies using binational samples found that US residence was associated with increased odds of cervical cancer screening (10,11). Both studies, however, had low participation rates and were limited to women aged 40 years or older. In *Healthy Border 2010*, the United States-Mexico Border Health Commission set goals for a 20% reduction in cervical cancer mortality for the Mexico border population and a 30% reduction in cervical cancer mortality for the US border population (12). To achieve this goal, Pap testing coupled with appropriate follow-up of abnormal Pap tests must increase on both sides of the border.

In countries with no organized screening programs, prenatal care offers an opportunity for women who typically have minimal contact with a health care provider to get a Pap test at least once in their lifetime. We compared the prevalence of lifetime cervical cancer screening and identified predictive factors among women on each side of the US-Mexico border who recently gave birth.

## Methods

### Data collection

The data used in this analysis were collected as part of the Brownsville-Matamoros Sister City Project for Women's Health (BMSCP), which began in the US-Mexico border sister cities of Matamoros, Tamaulipas, Mexico, and Brownsville, Cameron County, Texas, and was subsequently expanded to encompass all of Cameron County (Figure). The study used a stratified systematic cluster sampling probability design to select women who delivered live infants in Matamoros and Cameron County. Strata consisted of hospitals with 100 deliveries per year or more in either locality. Within each stratum, specific days were selected by using systematic sampling, and every woman who gave birth on selected days (within a cluster of days) was included in the sample. Of the 999 women sampled on selected days from August 21 through November 9, 2005, 947 (95%) completed interviews. The BMSCP pilot project was reviewed for human subject concerns by the Centers for Disease Control and Prevention (CDC) and was determined to be "nonresearch" or public health practice. A more thorough description of the data collection and other aspects of the BMSCP is provided elsewhere (13).

**Figure.**
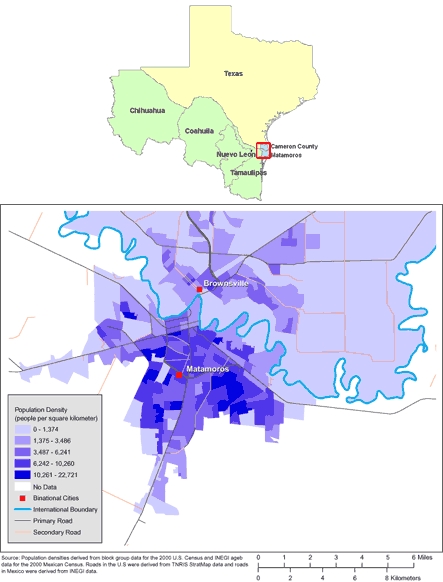
Maps of the US-Mexico Border Region (Top) and of Brownsville, Texas, and Matamoros, Tamaulipas, Mexico (Bottom). (The authors thank Allison Abell Banicki of the Office of Border Health, Texas Department of State Health Services, for creating the map of the Texas-Mexico border states and thank Jean W. Parcher, Sylvia N. Wilson, and the United States Geological Survey [USGS] for providing the map of population density in Brownsville and Matamoros.)

### Measures

Our 2 outcome variables of interest were ever having had a Pap test and having had a Pap test within the past 3 years. To assess whether women had ever had a Pap test, respondents were asked, "Have you ever had a Pap smear test?" Women who responded yes were then asked, "How long has it been since your most recent Pap smear?" Response options included less than 1 year, 1 to 2 years, 2 to 3 years, and 3 to 5 years. Women who did not respond to either question were coded as missing and excluded from the analyses. The final unweighted sample size was 941 responses distributed between Matamoros (n = 488) and Cameron County (n = 453).

Respondents who reported receiving at least 1 Pap test were asked, "Why did you have your most recent Pap smear test?" The question was open-ended, and responses were categorized into 1 or more of 11 preset responses: "consultation for family planning services," "consultation for pregnancy test," "to check health before getting pregnant," "checkup during pregnancy," "routine checkup," "because the doctor sent me," "because there was a campaign or promotion," "because it was about time," "gynecological symptoms or STD check," "disease of the cervix," and "other."

Health behavior was measured as a composite of 5 variables. One variable was a measure for risky behaviors for HIV or sexually transmitted diseases (STDs) (respondents who indicated that they had *not* participated in any of the following behaviors: intravenous drug use in the past year; treated for a "sexually transmitted disease," "sexually transmitted infection," or "venereal disease" in the past year; and more than 2 sex partners in the past year). Three variables were defined for the 3 months before the woman's pregnancy: walking for at least 10 minutes daily in a usual week, having had an HIV test during the most recent pregnancy, and seatbelt use. The last variable was consumption of nutritious foods, defined by at least daily consumption of fruits, green salad, or vegetables during the 3 months before getting pregnant. Positive healthy behaviors were summed and coded to identify respondents with 1 or 2 healthy behaviors, 3 healthy behaviors, or 4 or 5 healthy behaviors.

### Data analysis

We weighted the data to account for probability of selection, population noncoverage, hospital noncoverage, and nonresponse. The complex survey design was taken into account by using SUDAAN Release 9.01 (RTI International, Research Triangle Park, North Carolina). We analyzed data by place of residence and for the combined border region. Bivariate and multivariate analyses were conducted for both outcomes (ever and recent Pap test) with nearly identical results. Given these similar findings, we report only results for ever having had a Pap test. Differences in the prevalence of the outcome variables between women who delivered in Matamoros and women who delivered in Cameron County were assessed using a χ^2^ test for independence. Statistical significance was set at *P* < .05. Differences could not be assessed between the individual sides of the US-Mexico border and the combined data because the combined data were a composite of the data from individual sides of the US-Mexico border and observations were not independent.

Logistic regression was used to quantify the difference in the odds of the outcome variables by respondent characteristics. Variables that were statistically significant in the bivariate analyses were included in the multivariable logistic regression models. Variables that approached significance (*P* > .05 but < .10) were also included in the logistic regression model to account for other potential sources of variance and confounders. Only variables with at least 30 cases unweighted per level were considered in the logistic regression analysis. Models were estimated for Cameron County and Matamoros separately and for the combined sample.

## Results

The weighted prevalence of ever having had a Pap test was 62.1% among women who lived in Matamoros, compared with 95.7% among women who lived in Cameron County. The weighted prevalence of having had a Pap test in the previous 3 years was similar at 58.0% and 94.8%, respectively (Table 1).

Women primarily delivered in their country of residence. Nearly all Matamoros residents completed the interview in Spanish, compared with a nearly even distribution between respondents who completed the interview in Spanish or English in Cameron County. Of the total sample, 5% of respondents had an ethnicity other than Hispanic (Table 1).

Women who lived in Matamoros but delivered in the United States were more likely to have had a Pap test (88.8%) compared with women who lived in Matamoros and delivered in Mexico (60.7%) (Table 2). In the overall border region, 95.6% of women who delivered in the United States reported having had a Pap test compared with 60.6% of the women who delivered in Mexico. Increasing age was consistently associated with increased likelihood of having had a Pap test.

Maternal birthplace in Mexico was associated with a lower likelihood of ever having had a Pap test among residents of  Cameron County and the overall border region. In Cameron County, the prevalence of ever having had a Pap test among women born in Mexico was approximately 5 percentage points lower than that among women born in the United States. This disparity was nearly 30 percentage points in the overall border region.

Women with higher levels of education (≥12 years) in each county and overall were more likely to have had a Pap test. In Matamoros and the overall border region, increasing gravidity was associated with an increase in the prevalence of ever having had a Pap test. Differences between primigravid women and women with 5 or more pregnancies were among the largest of any reported.

In Cameron County and the overall border region, women who received any prenatal care were more likely to have had a Pap test compared with women who had no prenatal care. In Cameron County, the prevalence of ever having had a Pap test exceeded 95% among women receiving prenatal care but was 32.7% among women who received no prenatal care. In the overall border region, a similar pattern was observed.

In Matamoros and the overall border region, respondents with 4 or 5 healthy behaviors had higher rates of ever having had a Pap test. In Matamoros, the prevalence of ever having had a Pap test was approximately 18 percentage points higher for women with 4 to 5 healthy behaviors than for women with fewer healthy behaviors. In the overall border region, the difference between these groups was slightly greater, approximately 22 percentage points.

### Adjusted odds of ever having had a Pap test

After adjusting for other sources of variance, the disparity in Pap test prevalence persisted between the Cameron County and Matamoros (Table 3). In the multivariate analysis, women who resided in Cameron County had increased odds of ever having had a Pap test (adjusted odds ratio [AOR], 7.41; 95% confidence interval [CI], 4.07-13.48).

In Cameron County, women aged 19 years and younger had lower odds (AOR, 0.32; 95% CI, 0.10-0.96) of ever having had a Pap test compared with women aged 25 to 29 years (Table 3). In Matamoros, women aged 30 to 43 years had 2.64 (95% CI, 1.37-5.10) times higher odds of ever having had a Pap test compared with women aged 25 to 29 years, which was similar to the increase in the odds (AOR, 2.73; 95% CI, 1.49-5.01) in the overall border region. In the overall border region, women younger than 25 had reduced odds of ever having had a Pap test.

Education level was associated with the odds of ever having had a Pap test in the Matamoros and overall border region models (Table 3). In Matamoros and overall, the odds of having had a Pap test were lower for women with 8 to 12 years of education but no diploma, and for women with less than 8 years of education than for women who had 12 years of education or more with a diploma.

In the Matamoros multivariate model, compared with respondents who experienced only 1 pregnancy, experiencing 2 to 4 pregnancies increased the odds of ever having had a Pap test by 6.33 times, and experiencing 5 or more pregnancies was associated with a 9.74 times increase in the odds of ever having had a Pap test. A similar pattern was found in the combined analysis. Place of delivery and prenatal care did not have an adequate sample size for inclusion in the multivariate analysis.

In the multivariate model for Matamoros, among respondents with 4 or 5 healthy behaviors, the odds of ever having had a Pap test were 89% higher than for respondents with 1 or 2 healthy behaviors. In the multivariate model for the overall border region, among respondents with 4 or 5 healthy behaviors, the odds of ever having had a Pap test were 78% higher than for respondents with 1 or 2 healthy behaviors.

### Reason for having had a Pap test

In Matamoros, a routine checkup (26.8%) and provider referral (25.5%) were the 2 most commonly cited reasons women gave for getting their most recent screening test (Table 4); 12.7% cited a checkup during pregnancy. In Cameron County, nearly three-quarters (73.8%) of respondents indicated that a checkup during pregnancy was their screening opportunity.

## Discussion

This study identifies a large disparity in Pap testing between women who delivered in Matamoros and those who delivered in Cameron County and suggests that prenatal care is a valuable opportunity to increase Pap test coverage. Rates of Pap testing in Matamoros were similar to rates reported in other Mexican studies (1,14,15). Conversely, in Cameron County, the rates reported in this study exceeded previously reported overall rates in Texas and rates specific to the Texas-Mexico border, most likely because the Cameron County women in our survey had just delivered and almost three-quarters of them had had a Pap test during prenatal visits (16,17).

Pap testing has been a routine part of prenatal care in the United States since the early 1980s (18). Interaction with a health care provider during pregnancy may present an opportunity for increased use of preventive services, including cervical cancer screening and referral. By focusing on women who recently delivered a live infant, this study explores cervical cancer screening in a subgroup in which the lifetime screening prevalence is expected to be much higher. Previous studies have found lifetime Pap test screening to be a cost-effective method to reduce disease burden, especially in resource-limited settings (19-21). These data suggest that an opportunity to increase lifetime Pap test prevalence is being missed in Matamoros.

Whether prenatal care represents the best opportunity to increase Pap testing is a source of controversy. Nygård et al recently evaluated the role of prenatal care in improving Pap test rates in Norway (22). They concluded that this strategy needs to consider country-specific factors such as the age at which the incidence of precancerous lesions peaks, the age at which human papillomavirus (HPV) prevalence peaks, the mean age of pregnancy, the accuracy of the prenatal Pap test to diagnose underlying preinvasive lesions, and the impact this strategy may have on coverage compared with other strategies (22).

Whether cervical cancer screening needs to be cytology-based is another heavily debated topic in Latin America. Cytology-based screening presents challenges in resource-limited settings, but recent research has suggested that DNA testing for HPV, which uses an assay system, can improve access to cervical cancer screening in resource-limited settings and, because the specimen can be self-collected, this type of test may reduce the patient anxiety and apprehension sometimes associated with Pap testing. However, HPV DNA testing is expensive. On the other hand, visual inspection with acetic acid (VIA) is more affordable for a developing country and is considered more accurate than Pap tests (23,24). Although VIA may yield some false-positives and lead to overtreatment, VIA offers an immediate result, so treatment can be initiated right away.

Age and gravidity were positively associated with Pap testing. These findings are expected when considering the cervical cancer screening guidance in each country. In Mexico, routine cervical cancer screening begins at age 25 and should be conducted every 3 years (1). In the United States, most guidelines recommend that a Pap test should begin within 3 years after initiating sexual activity or by age 21, whichever is earlier (25,26). The most cost-effective strategies to reduce the burden of cervical cancer in low-resource settings is to offer 1 or 2 screenings starting at age 35 (21). A recent economic analysis in Mexico concluded that HPV vaccination (when affordable vaccine is available) at age 12 and a combination of Pap and HPV screening for women aged 25 to 64 years may be more beneficial than Pap testing alone (27).

Our findings demonstrate a need for binational collaboration to support healthy behaviors. Women in this study who practiced several healthy behaviors had higher screening rates. This finding suggests that a coordinated binational campaign supporting healthy behaviors would not only reduce preventable illness and death but also could influence cervical cancer screening rates.

Hispanic women in Cameron County were more likely to have had a Pap test compared with non-Hispanic women. This finding is in contrast to previous studies that consistently reported a lower prevalence of cervical cancer screening among Hispanic women, especially among Mexican-origin Hispanic women (16,28-30). There are several possible explanations for this finding. Barriers to cervical cancer screening are perceived pain, lack of knowledge of the test, and not knowing where to go (16,28,29). In at least 1 study, Hispanic women cited physician sex and insensitivity to patient needs as barriers to cervical cancer screening (28). With increasing numbers of women in obstetric practice (31-33), this barrier may not be as significant in the prenatal population and may contribute to the increased prevalence of lifetime Pap testing in this study population. Another possible explanation is that the ethnic concentration of Hispanic women in Cameron County, 88.9%, provides increased social support and reduced barriers. Further research is needed to confirm and examine possible causes for this finding.

Failure to screen for cervical cancer increases the likelihood of late-stage diagnosis, the risk of mortality, and health care costs. In Mexico, despite the availability of cervical cancer screening for more than 35 years, less than 13% of preventable cases have been averted (4). Women who participate in prenatal care have increased interaction with the health care system. This increase in interaction presents an opportunity to increase lifetime cervical cancer screening prevalence by ensuring that all women receive screening during prenatal care. Increased efforts may be needed to discuss with Mexico's policy makers, physicians, and public health community whether prenatal care is the best opportunity to increase lifetime Pap testing prevalence and help to achieve the *Healthy Border 2010* goal of a 20% reduction in cancer mortality (12).

## Acknowledgments

The BMSCP was funded through the Division of Reproductive Health and the Office of Global Health Promotion at the National Center for Chronic Disease Prevention and Health Promotion, CDC, under a cooperative agreement with the United States-Mexico Border Health Association, No. U65 CCU 623699-01-2, and through interagency personnel agreements with the University of Texas at Brownsville, Texas Southmost College, and the University of Texas-Houston School of Public Health, Brownsville Regional Campus. In-kind project support was provided by the Division of Health Examination Statistics at the National Center for Health Statistics, CDC; the Texas Department of State Health Services, Region 11; the Secretariat of Health, Tamaulipas; and the Mexican Institute of Social Security, Tamaulipas.

Support from the following local, regional, and national institutions was critical to the project: the National Center for Gender Equity and Reproductive Health, Mexican Health Secretariat; National Center for Epidemiologic Surveillance and Disease Control, Mexican Health Secretariat; National Center for Health Promotion, Mexican Health Secretariat; National Institute of Statistics, Geography and Informatics, Tamaulipas; Civil Registry, Tamaulipas; Institute for Social Security and Services for State Workers, Tamaulipas; Secretariat of Health, Jurisdiction III, Tamaulipas; Texas Department of State Health Services, Region 11 and Office of Border Health; City of Brownsville Department of Public Health; Cameron County Health Department; Valley Baptist Medical Center in Harlingen; Valley Baptist Medical Center in Brownsville; Valley Regional Medical Center; Harlingen Medical Center; Cameron Park Cultural Center; Brownsville Community Health Center; General Hospital of Matamoros; Dr. Alfredo Pumarejo Lafaurie; Mexican Institute of Social Security General Hospital, Zone #13, Matamoros; Dr Manuel F. Rodríguez Brayda Clinical Hospital, Matamoros; Hospital Guadalupe; Matamoros Center of Family Orientation; Medical Center of Surgical Specialties of Matamoros, and the United States-Mexico Border Health Commission. We thank the National Center for Gender Equity and Reproductive Health, Secretariat of Health, Mexico, for review of this manuscript and the United States-Mexico Border Health Commission for providing the English-to-Spanish translation.

## Figures and Tables

**Table 1:** Characteristics of Women Who Gave Birth in the US-Mexico Border Region, Brownsville-Matamoros Sister City Project for Women's Health, 2005

**Characteristics**	Place of Residence				*P* value^b^	Total Sample	

	Matamoros		Cameron County		
	
	Unweighted Frequency (n = 488)^a^	Weighted Frequency (%) (n = 2,758)^a^	Unweighted Frequency (n = 453)^a^	Weighted Frequency (%) (n = 2,310)^a^		Unweighted Frequency (n = 941)	Weighted Frequency (%) (n = 5,068)
**Ever had a Pap test**							
Yes	304	1,713 (62.1)	434	2,212 (95.7)	<.001	738	3,925 (77.4)
No	184	1,045 (37.9)	19	99 (4.3)		203	1,143 (22.6)
**Had a Pap test within past 3 years**							
Yes	284	1,600 (58.0)	430	2,191 (94.8)	<.001^c^	714	3,791 (74.8)
No	204	1,158 (42.0)	23	119 (5.2)		227	1,277 (25.2)
**Place of delivery**							
United States	27	139 (5.0)	452	2,305 (99.8)	<.001	479	2,444 (48.2)
Mexico	461	2,618 (95.0)	ND^d^	5 (0.2)		462	2,624 (51.8)
**Ethnicity**							
Hispanic	488	2,758 (100.0)	393	2,003 (88.9)	<.001	881	4,761 (95.0)
Non-Hispanic	ND^e^	ND^e^	49	250 (11.1)		49	250 (5.0)
**Age, y**							
≤19	94	532 (19.3)	66	336 (14.5)	.07	160	868 (17.1)
20-24	154	871 (31.6)	140	715 (30.9)		294	1,586 (31.3)
25-29	135	763 (27.7)	117	597 (25.8)		252	1,359 (26.8)
30-43	105	592 (21.5)	130	662 (28.7)		235	1,254 (24.7)
**Marital status**							
Not Married	46	258 (9.4)	119	607 (26.4)	<.001	165	864 (17.1)
Married	440	2,489 (90.6)	332	1,694 (73.6)		772	4,182 (82.9)
**Education level, y**							
<8	156	883 (32.0)	55	280 (12.2)	<.001	211	1,163 (23.0)
8-12 (no diploma)	248	1,404 (50.9)	168	858 (37.3)		416	2,262 (44.7)
≥12 (diploma)	84	471 (17.0)	228	1,163 (50.5)		312	1,633 (32.3)
**Place of birth**							
United States	ND^d^	11	251	1,280 (56.4)	<.001	253	1,291 (25.8)
Mexico	483	2,729 (98.9)	194	990 (43.6)		677	3,719 (74.2)
**Language spoken during interview**							
English	3	17 (0.6)	234	1,190 (51.5)	<.001	237	1,207 (23.8)
Spanish	485	2,741 (99.4)	219	1,120 (48.5)		704	3,861 (76.2)
**Smoked cigarettes^f^**							
Yes	24	135 (4.9)	36	184 (8.0)	.01	60	319 (6.3)
No	464	2,622 (95.1)	416	2,122 (92.0)		880	4,744 (93.7)
**Age at first sexual intercourse, y**							
<16	100	565 (20.5)	86	438 (19.1)	.29	186	1,004 (19.9)
16-17	128	726 (26.3)	136	693 (30.2)		264	1,419 (28.1)
≥18	260	1,467 (53.2)	228	1,164 (50.8)		488	2,630 (52.0)
**Health care coverage**							
Coverage at any time	357	2,026 (73.6)	315	1,605 (69.6)	.08	672	3,631 (71.8)
No coverage	130	726 (26.4)	137	700 (30.4)		267	1,427 (28.2)
**Gravidity**							
1	172	971 (35.2)	132	673 (29.1)	.09	304	1,644 (32.4)
2-4	279	1,578 (57.2)	276	1,407 (60.9)		555	2,985 (58.9)
≥5	37	208 (7.5)	45	230 (10.0)		82	439 (8.7)
**Entry into prenatal care**							
1st trimester	216	1,219 (44.9)	275	1,403 (61.6)	<.001	491	2,621 (52.5)
2nd trimester	228	1,292 (47.6)	152	776 (34.1)		380	2,067 (41.4)
3rd trimester	19	107 (3.9)	16	81 (3.6)		35	189 (3.8)
No prenatal care	17	95 (3.5)	ND^d^	16 (0.7)		20	111 (2.2)
**Healthy behaviors^g^**							
1 or 2	76	430 (15.6)	24	123 (5.3)	<.001	100	553 (10.9)
3	156	882 (32.0)	61	311 (13.5)		217	1,193 (23.5)
4 or 5	256	1,446 (52.4)	368	1,876 (81.2)		624	3,322 (65.5)

Abbreviations: Pap, Papanicolaou; ND, not determined.

a Columns do not all total to number in sample size because of missing data.

b χ^2^ test used to determine statistical differences.

c Values for having had a Pap test in the last 3 years were so similar to values for having ever had a Pap test that only the latter were analyzed.

d Cell values of <3 were not used to calculate weighted frequencies.

e In Matamoros, all women are considered to be of Hispanic ethnicity. Therefore, no data are reported for non-Hispanic ethnicity and the χ^2^ test was not calculated.

f Respondents who smoked 100 cigarettes in the past 2 years or who smoked any cigarettes on an average day 3 months before this pregnancy.

g Healthy behaviors were defined as 1)* not* participating in any of the following behaviors: intravenous drug use in the past year, treated for a sexually transmitted infection in the past year, and more than 2 sex partners in the past year; 2) walking for at least 10 minutes daily in a usual week in the 3 months before pregnancy; 3) having had an HIV test during the most recent pregnancy; 4) seatbelt use; and 5) consumption of nutritious foods during the 3 months before getting pregnant.

**Table 2:** Prevalence of Lifetime Pap Test Screening Among Women Who Gave Birth in the US-Mexico Border Region, Brownsville-Matamoros Sister City Project for Women's Health, 2005

**Characteristic**	Place of Residence				Total Sample	

	Matamoros		Cameron County			

	Weighted Percentage(95% CI)	*P* value^a^	Weighted Percentage(95% CI)	*P* value^a^	Weighted Percentage(95% CI)	*P* value^a^
**Place of delivery**						
United States	88.8 (78.5-99.0)	.001	96.0 (94.6-97.3)	.27	95.6 (94.2-96.9)	<.001
Mexico	60.7 (56.0-65.4)		0		60.6 (55.8-65.3)	
**Ethnicity**						
Hispanic	62.1 (57.6-66.7)	ND^b^	97.2 (95.6-98.7)	.03	76.9 (73.9-79.9)	.12
Non-Hispanic	ND^b^		85.3 (75.7-94.9)		85.3 (75.7-94.9)	
**Age, y**						
≤19	39.2 (31.3-47.0)	<.001	87.6 (82.0-93.2)	<.001	57.9 (51.5-64.4)	<.001
20-24	47.3 (40.2-54.4)		95.6 (92.3-98.9)		69.1 (63.7-74.4)	
25-29	73.1 (66.3-79.9)		96.6 (93.9-99.3)		83.4 (79.3-87.5)	
30-43	90.4 (86.9-93.9)		99.2 (97.8-100.0)		95.1 (93.1-97.0)	
**Marital status**						
Not married	67.1 (53.5-80.7)	.45	96.6 (93.8-99.5)	.43	87.8 (82.5-93.1)	<.001
Married	61.7 (56.8-66.5)		95.4 (93.9-96.7)		75.3 (72.0-78.6)	
**Education level, y**						
<8	66.5 (59.0-74.0)	.002	96.3 (91.7-100.0)	.03	73.7 (67.8-79.5)	<.001
8-12 (no diploma)	56.3 (51.0-61.7)		92.7 (89.5-95.9)		70.1 (65.9-74.4)	
≥12 (diploma)	71.2 (62.7-79.6)		97.8 (96.2-99.4)		90.1 (87.0-93.2)	
**Maternal place of birth**						
United States	50.0 (0-100.0)	.73	98.0 (96.7-99.3)	.007	97.6 (96.1-99.0)	<.001
Mexico	62.1 (57.5-66.8)		92.7 (89.3-96.0)		70.3 (66.5-74.1)	
**Language spoken during interview**						
English	66.8 (19.1-100.0)	.85	97.8 (96.4-99.3)	.007	97.4 (95.7-99.0)	<.001
Spanish	62.1 (57.5-66.7)		93.5 (90.0-96.1)		71.2 (67.5-74.9)	
**Smoked cigarettes^c^**						
Yes	70.5 (52.3-88.8)	.37	94.3 (87.7-100.0)	.67	84.2 (75.5-93.0)	.13
No	61.7 (57.1-66.3)		95.8 (94.4-97.3)		77.0 (74.0-79.9)	
**Age at first sexual intercourse, y**						
<16	66.0 (56.1-75.8)	.44	95.4 (91.4-99.4)	.65	78.8 (73.1-84.5)	.53
16-17	63.2 (54.8-71.6)		94.6 (91.8-97.4)		78.6 (73.9-83.2)	
≥18	60.1 (54.0-66.2)		96.5 (94.2-98.7)		76.2 (72.3-80.1)	
**Health care coverage**						
Coverage at any time	61.0 (55.6-66.3)	.18	97.7 (96.5-99.0)	.02	77.2 (73.8-80.7)	.70
No coverage	65.8 (59.8-71.8)		91.1 (86.1-96.1)		78.2 (74.1-82.4)	
**Gravidity**						
1	31.7 (25.8-37.6)	<.001	93.1 (89.3-96.9)	.13	56.8 (51.6-62.0)	<.001
2-4	77.3 (72.7-81.9)		96.6 (95.1-98.2)		86.4 (83.9-89.0)	
≥5	89.1 (81.2-96.9)		97.8 (94.0-100.0)		93.6 (89.2-98.1)	
**Entry into prenatal care (current pregnancy)**						
1st trimester	66.0 (59.1-73.0)	.21	95.9 (93.8-98.1)	<.001	82.0 (78.3-85.7)	.001
2nd trimester	60.4 (54.8-66.0)		96.0 (93.6-98.3)		73.7 (69.7-77.8)	
3rd trimester	47.5 (31.5-63.5)		100.0		70.1 (58.7-81.5)	
No prenatal care	58.6 (32.4-84.8)		32.7 (0-79.2)		55.0 (31.6-78.4)	
**Healthy behaviors^d^**						
1 or 2	53.6 (41.5-65.8)	<.001	91.1 (80.6-100.0)	.63	62.0 (51.3-72.7)	<.001
3	52.4 (46.2-58.6)		95.0 (90.5-99.6)		63.5 (58.6-68.4)	
4 or 5	70.6 (64.7-76.2)		96.2 (94.4-97.9)		85.0 (82.2-87.8)	

Abbreviations: Pap, Papanicolaou; CI, confidence interval; ND, not determined.

a χ^2^ test used to determine significance.

b In Matamoros, all women are considered to be of Hispanic ethnicity. Therefore, no data are reported for non-Hispanic ethnicity and the Χ^2^ test was not calculated.

c Respondents who smoked 100 cigarettes in the past 2 years or who smoked any cigarettes on an average day 3 months before this pregnancy.

d Healthy behaviors were defined as 1)* not* participating in any of the following behaviors: intravenous drug use in the past year, treated for a sexually transmitted infection in the past year, and more than 2 sex partners in the past year; 2) walking for at least 10 minutes daily in a usual week in the 3 months before pregnancy; 3) having had an HIV test during the most recent pregnancy; 4) seatbelt use; and 5) consumption of nutritious foods during the 3 months before getting pregnant.

**Table 3:** Adjusted Odds Ratios of Lifetime Pap Test Screening Among Women Who Gave Birth in the US-Mexico Border Region, Brownsville-Matamoros Sister City Project for Women's Health, 2005

**Characteristic**	Matamoros (n = 488) AOR (95% CI)	Cameron County (n = 453) AOR (95% CI)	Matamoros and Cameron County (n = 941) AOR (95% CI)
**Place of residence^a^**			
United States	ND^b^	ND^b^	7.41 (4.07-13.48)
Mexico	ND^b^	ND^b^	1.00
**Ethnicity**			
Hispanic	ND^c^	4.41 (1.28-15.19)	ND^b^
Non-Hispanic	ND^c^	1.00	ND^b^
**Marital status**			
Not married	ND^b^	ND^b^	2.59 (1.40-4.81)
Married	ND^b^	ND^b^	1.00
**Age, y**			
≤19	0.76 (0.42-1.39)	0.32 (0.10-0.96)	0.50 (0.30-0.82)
20-24	0.44 (0.25-0.78)	1.72 (0.39-7.62)	0.49 (0.30-0.81)
25-29	1.00	1.00	1.00
30-43	2.64 (1.37-5.10)	4.71 (0.71-31.42)	2.73 (1.49-5.01)
**Education level, y**			
<8	0.55 (0.32-0.96)	3.38 (0.41-27.65)	0.65 (0.40-1.04)
8-12 (no diploma)	0.44 (0.27-0.70)	0.68 (0.24-1.94)	0.50 (0.32-0.81)
≥12 (with diploma)	1.00	1.00	1.00
**Maternal place of birth**			
United States	ND^b^	1.00	1.00
Mexico	ND^b^	0.45 (0.10-2.10)	0.42 (0.14-1.26)
**Language spoken during interview**			
English	ND^b^	1.00	1.00
Spanish	ND^b^	0.93 (0.21-4.12)	0.81 (0.30-2.21)
**Health care coverage**			
Coverage at any time	ND^b^	3.47 (0.88-13.65)	ND^b^
No coverage	ND^b^	1.00	ND^b^
**Gravidity**			
1	1.00	ND^b^	1.00
2-4	6.33 (3.95-10.20)	ND^b^	5.13 (3.42-7.69)
≥5	9.74 (2.68-35.40)	ND^b^	6.82 (2.15-21.69)
**Healthy behaviors^d^**			
1 or 2	1.00	ND^b^	1.00
3	0.97 (0.63-1.50)	ND^b^	1.04 (0.71-1.53)
4 or 5	1.89 (1.24-2.88)	ND^b^	1.78 (1.18-2.68)

Abbreviations: Pap, Papanicolaou; AOR, adjusted odds ratio; CI, confidence interval; ND, not determined.

a Place of residence was used to stratify the residence-specific models and therefore could not be included as a correlate in the model. However, place of residence was included in the Matamoros and Cameron County model.

b Each column represents a separate logistic regression model and all variables included in the model. Variables that satisfy the eligibility criteria for inclusion in 1 model may not for another model. ND denotes that a variable did not meet the criteria for inclusion in that model, but did for 1 or more of the other models.

c In Matamoros, all women are considered to be of Hispanic ethnicity. Therefore, no data are reported for non-Hispanic ethnicity and the χ^2^ test was not calculated.

d Healthy behaviors were defined as 1) *not* participating in any of the following behaviors: intravenous drug use in the past year, treated for a sexually transmitted infection in the past year, and more than 2 sex partners in the past year; 2) walking for at least 10 minutes daily in a usual week in the 3 months before pregnancy; 3) having had an HIV test during the most recent pregnancy; 4) seatbelt use; and 5) consumption of nutritious foods during the 3 months before getting pregnant.

**Table 4:** Reason for Most Recent Pap Test Among Women Who Gave Birth in the US-Mexico Border Region, Brownsville-Matamoros Sister City Project for Women's Health, 2005

Reason	Place of Residence		Total Sample Weighted Percentage (95% CI)

	Matamoros Weighted Percentage (95% CI)	Cameron County Weighted Percentage (95% CI)	
Consultation for family planning services	2.1 (0.4-3.8)	1.5 (0.3-2.6)	1.8 (0.8-2.7)
Consultation for pregnancy test	0.7 (0-1.7)	0	0.3 (0-0.7)
To check health before pregnancy	2.5 (0.7-4.3)	0.5 (0-1.2)	1.4 (0.5-2.2)
Checkup during pregnancy	12.7 (8.9-16.5)	73.8 (69.6-77.9)	47.1 (43.7-50.5)
Routine checkup	26.8 (21.7-31.9)	12.3 (9.1-15.5)	18.7 (15.8-21.5)
Because the doctor sent me	25.5 (20.4-30.5)	4.2 (2.2-6.2)	13.5 (10.9-16.0)
Because there was a campaign or promotion	13.8 (9.8-17.8)	0.2 (0-0.7)	6.2 (4.3-8.0)
Because it was about time	10.2 (6.7-13.7)	7.5 (4.9-10.0)	8.7 (6.6-10.7)
Had STD/GYN symptoms	2.5 (0.7-4.3)	0	1.1 (0.3-1.9)
Disease of cervix	3.2 (1.1-5.3)	0	1.4 (0.5-2.3)

Abbreviations: Pap, Papanicolaou; CI, confidence interval; STD, sexually transmitted disease; GYN, gynecologic.
